# Atypical Reaction Media and Organized Systems for the Synthesis of Low-Substitution Sugar Esters

**DOI:** 10.3389/fchem.2019.00587

**Published:** 2019-09-23

**Authors:** Sidrine Kerthy Koumba Ibinga, Jean-François Fabre, Raphaël Bikanga, Zéphirin Mouloungui

**Affiliations:** ^1^Laboratoire de Chimie Agro-industrielle, Université de Toulouse, INRA, Toulouse, France; ^2^Laboratoire des Substances Naturelles et de Synthèse Organo-Métallique, LASNSOM, Université des Sciences et Techniques de Masuku, Franceville, Gabon

**Keywords:** sugars, organized media, sugar fatty acid esters, degree of substitution, ionic liquids, deep eutectic solvent, emulsion

## Abstract

Sugar esters are non-ionic surfactants with amphiphilic properties of interest for the formulation of various products in the fields of detergents, foods, medicines, pharmaceuticals, agriculture, and cosmetics. The properties of sugar esters depend on their degree of substitution (we consider degrees of substitution between 1 and 3 here) which guides their use. Sugar esters are biodegradable and non-toxic, and the demand for these compounds is high and continuing to increase. Indeed, interest in these compounds stems from the natural origin of the raw materials, the synthetic processes involved and the performance of the final product. The choice of reaction medium is crucial, to facilitate contact between reactants and prevent hydrolysis of the products. In this review, we provide an overview of the processes and synthesis routes for sugar ester production, ionic liquids and deep eutectic solvent as non-usual media or with organized systems.

## Introduction

Sugars are renewable resources ideal for the development of chemical synthesis of new biocompatible and biodegradable sugar-based surfactants (Silvestre et al., 2010). Sugar fatty acid esters (SFAEs) are amphiphilic molecules with properties dependent on their degree of substitution (DS). DS is defined as the number of esterified hydroxyl groups. Previous reviews have reported the synthesis pathways, properties and potential applications of these compounds in the food, pharmaceutical, and cosmetic industries (Akoh, 2002; Zheng et al., 2015). Synthesis focuses on esters with a DS of 1–3 which have a number of interesting properties: hydrophilic-lipophilic balance (HLB), critical micelle concentration (CMC), surface tension, emulsifying stability (especially O/W and some W/O emulsions, multiple emulsions), foaming ability (Liang et al., 2018; Ma et al., 2018), biological activities, and thermal properties. Sucrose esters (SEs) are the most widely used; those with a DS of 1–4 are hydrophilic, whereas those with a DS of 5–8 are lipophilic. Sucrose esters with a low DS have an HLB value of about 5–16 and are most suitable for use in O/W emulsions. Hydrophilic sucrose esters are digestible and absorbable (DS 1–3), antimicrobial (DS 1–2), and can be used as emulsifiers, wetting or dispersion agents, solubilization, stabilization, and antimicrobial agents (Szuts and Szabó-Révész, 2012; Zheng et al., 2015).

The HLB, CMC, and surface tensions of the sucrose esters are reported (Soultani et al., 2003; Zhang et al., 2014; Zheng et al., 2015). SFAEs are odorless, non-toxic, biodegradable do not irritate the skin (Ferrer et al., 1999; Madsen et al., 2001; Plat and Linhardt, 2001). Some SFAEs have been shown to have insecticidal, antibacterial, and antitumor activities (Okabe et al., 1999; Watanabe et al., 2000; Puterka et al., 2003; Ferrer et al., 2005; Zhang et al., 2015; AlFindee et al., 2018). These compounds are stable at temperatures of up to 120–130°C, with total decomposition generally occurring at 260°C.

SFAEs are synthesized by esterification or transesterification from carbohydrates and fat in the presence of a chemical or enzymatic catalyst. The synthesis of these compounds is subject to several difficulties, such as low reactivity due to the immiscibility of the raw materials, regioselectivity and control over the DS which may orient and condition the synthesis of the desired products and their properties. SFAEs have long been obtained in organic solvents, such as dimethylsufoxyde, dimethylformamide and pyridine, in which both sugars and fats are soluble (Piccicuto et al., 2001). However, these organic solvents are toxic, non-volatile, and incompatible with food applications. In this mini-review, we present three original experimental approaches for overcoming these problems. The first two approaches involve the synthesis of SFAEs with atypical solvents, such as ionic liquids (ILs) and deep eutectic solvents (DESs). ILs and DESs have been described as potential solvents for several reactions in the last 20 years (Yang and Huang, 2012; Nowicki and Muszynski, 2014; Megías-Sayago et al., 2016; Vanda et al., 2018). The third approach involves iterative micellization to drive the molecular organization of the particles and their reactivity (Claverie et al., 2004; Zhao et al., 2014).

## Ionic Liquids for the Synthesis of SFAEs

ILs and DESs have emerged as attractive green alternatives to organic solvents. Interest in these solvents has increased since 1985 (Seddon, 1997; Zhang et al., 2012; Paiva et al., 2014; Smith et al., 2014; Husson et al., 2018; Vanda et al., 2018) (Supplementary Materials). ILs are organic salts that melt below 100°C and are liquid at room temperature. They are often referred to as “designer solvents” because they are obtained by combining anions and cations. They are non-volatile and have good chemical and thermal stabilities. They can be used to dissolve polar, non-polar, inorganic, and polymeric organic compounds (Yang and Pan, 2005).

ILs have been used in SFAEs synthesis, to replace conventional solvents and overcome the problems of sugar solubility and the immiscibility of sugar and fat. The choice of ILs as solvents for the synthesis of SFAEs is based on their ability to dissolve carbohydrates (Liu et al., 2005; Zhao et al., 2008; Yang and Huang, 2012), which appears to be strongly related to the nature of the anion and the cation (Galonde et al., 2013). ILs containing the dicyanamide anion ([dca]) can dissolve up to 145 g/L glucose and 220 g/L sucrose at room temperature (Forsyth and MacFarlane, 2003; Liu et al., 2005) and have a low viscosity (MacFarlane et al., 2001). Reactions may be performed in the presence or absence of enzymes (Abdulmalek et al., 2012; Yang and Huang, 2012; Findrik et al., 2016). In chemical synthesis, ILs can act as both the catalyst and the solvent of the reaction (Forsyth et al., 2002; Lin et al., 2016). The choice of IL for enzymatic reactions is crucial, to ensure that the enzyme is not deactivated. Enzyme activity in ILs is limited by the viscosity of the medium, but this problem can be overcome by ultrasound irradiation (Lee et al., 2008).

Ganske et al. obtained glucose monoesters by using fatty acids and the corresponding vinyl esters as acyl donors in the presence of the *Candida antarctica* lipase B (CAL-B) immobilized on poly(ethylene glycol). No ester formation was observed with fatty acids in pure ILs, whereas, with vinyl esters, the rate conversion to esters was 30–35%. However, when reactions were performed with a mixture of IL and 40% *ter*-butanol, esters formed with both fatty acids (conversion rate of 64%) and vinyl esters (conversion rate of 90%), with monoester yields of 48 and 89%, respectively (Ganske and Bornscheuer, 2005). Findrik et al. showed that it was possible to react fatty acids with glucose in the same pure IL with a high conversion rate (Findrik et al., 2016). Liang et al. performed an enzymatic synthesis of glucose monopalmitate with 1-butyl-3-methylimidazolium trifluoromethanesulfonate ([Bmim][TfO-]) as the IL. Glucose was reacted with palmitic acid vinyl ester in the presence of CAL-B lipase as the catalyst. [Bmim][TfO-] dissolved more glucose when the enzyme was activated, and the solubility of glucose in ILs increased with temperature (Liang et al., 2012).

Mai et al. reported a preparative method for generating glucose monolaurate in a mixture of two ILs: 1-butyl-3-methylimidazolium trifluorosulfonate ([Bmim][TfO]) and 1-butyl-3-methylimidazolium bis(trifluoromethyl)sulfonylimide ([Bmim][Tf_2_N]). They used a supersaturated glucose solution (12g/L), with vinyl laurate as the acyl donor and *Candida antarctica* lipase B (Novozym 435) immobilized on macro-porous polyacrylate resin as the catalyst. Under optimal reaction conditions, they obtained a conversion yield of 96%. They also showed that the IL and enzyme could be recycled and reused for up to 10 cycles (Mai et al., 2014). Liu et al. synthesized sucrose dodecanoate with an immobilized lipase (Novozym 435) and an activated molecular sieve mixed with [BMIm][dca] (Liu et al., 2005).

Lin et al. described the synthesis of glucose monolaurate in ILs (Lin et al., 2015, 2016) with and without the use of enzymes. They studied the impact of the IL on SFAEs synthesis by enzymatic processes and they showed that better ester formation was achieved with ILs with a hydrophobic cation and a hydrophilic anion, such as [HMIm][TfO]. Tetraalkylammonium acetate was used as the solvent and chemical catalyst in enzyme-free reaction. A two-solvent system based on Tetrabutylammonium acetate ([Bu_4_N][Ac]) and 2-methyl-butanol allows a high glucose solubility (13 mM in 2-methyl-butanol vs. 173 mM in [Bu_4_N][Ac]) and a lower viscosity of the medium. The products obtained in the presence and absence of enzyme had similar retention times on HPLC, suggesting that specificity as retained. The regioselective reaction takes place in mild conditions and there is no need to eliminate water during the reaction. However, the products obtained in the presence of ILs were difficult to recover. A synthesis mechanism has been proposed for this reaction: in the presence of water, tetraalkylammonium ions (tetrabutyl, tetraethyl and tetramethyl) and acetates are dissolved. The acetates attack the oxygen in position 6 of glucose by nucleophilic substitution. The non-binding glucose doublet then attacks the carbonyl group of vinyl laurate and a tetrahedral intermediate is formed. This intermediate breaks down into glucose ester due to the provision of the leaving group, vinylate, by the activated acyl donor, the vinyl ester.

This work showed that it was possible to obtain SFAEs with a low DS from reactions in which ILs were used not only as the solvent, but also as a catalyst, in the presence or absence of an enzyme. In 2015, Zheng et al. wrote a book chapter about the synthesis of SFAEs, their physicochemical properties and their potential uses. They described chemical and enzymatic synthesis of sugar esters in organic solvents and ILs (Zheng et al., 2015). In the same year, the first synthesis of SFAEs with DES as the solvent was reported. In this review, we also consider the synthesis of sugar esters with DES as the solvent and in solvent-free emulsification processes.

## Synthesis of SFAEs in the Presence of DES

DESs are a new type of green solvent obtained by mixing two or more compounds to obtain a substance with a melting point lower than the melting points of each of the individual compounds. When the compounds used to prepare the DES are primary metabolites, such as amino acids, organic acids, sugars or choline derivatives, the resulting DESs are called Natural Deep Eutectic Solvents (NADES) (Dai et al., 2013). DESs and NADES can find applications in catalysis, organic synthesis, biotechnology and bioengineering, electrochemistry (Zhang et al., 2012; Smith et al., 2014; Mbous et al., 2017). DESs are prepared by complexing a quaternary ammonium salt, such as choline chloride, with a hydrogen bond donor, such as amine, alcohol, amide or carboxylic acid. DESs differ from ILs in the source of the raw materials and the chemical processes used for their preparation. DESs are easier to prepare and are obtained from readily available cheaper compounds. They are also considered to be more biodegradable, biocompatible and sustainable compounds than ILs. They may therefore be considered a useful alternative to ILs.

One of the most frequently encountered problems with the use of DESs as solvents is the high viscosity of the medium. Studies of the thermophysical properties of DESs (density, viscosity, polarity, conductivity) is an important area of investigation for the adaptation of DES preparations for the desired applications (Craveiro et al., 2016). Hayyan et al. studied the physical properties of a DES consisting of choline chloride (ChCl) and glucose (Glc). They found that, for ChCl:Glc ratios of 1:1, 1.5:1, 2:1, 2:1, and 2.5:1 by weight, the DES obtained was colorless liquid at room temperature. In the other conditions tested (ChCl:Glc ratios of 1:1.5, 1:2 and 1:2.5), the mixture was semi-solid. Viscosity and surface tension were lowest for a ratio of 2:1 by weight at 85°C. This study showed that DESs prepared from choline and glucose generally had a high viscosity, density and surface tension at room temperature (Hayyan et al., 2013). Zhao et al. reported the synthesis of SFAEs in a two-solvent mixture of DES and 2-methyl-2-butanol in the presence of lipozyme TLIM (*Thermomyces lanuginose* lipase) as the catalyst. They obtained glucose monoester yields of <15%, probably due to the properties of the solvent (Zhao et al., 2016).

Pöhnlein et al. studied the enzymatic synthesis of glucose hexanoate in various DESs (Pöhnlein et al., 2015). The DESs were prepared by mixing ammonium salts and the hydrogen donor in various proportions at 100°C until a liquid mixture was obtained. The various reagents (glucose: 1.38 mmol and vinyl hexanoate: 2.72 mmol) were dissolved in 3.5 mL DES. The enzyme was then added and the reaction mixture was incubated for three days at 70°C. DES has the advantage of being a good solvent for both substrates. As the DES acts as both substrate and solvent, the addition of more sugar is unnecessary. Glucose 6-O-hexanoate with a DS of 1 was obtained as the product.

## Synthesis of SFAEs in Organized Media

Acyl transfer reactions have also been performed in an organized system, using a surfactant for the synthesis of sugar fatty acid esters. The micellization steps for the synthesis of the SFAEs have been studied, to increase the contact area between the reagents.

**Figure 1:**
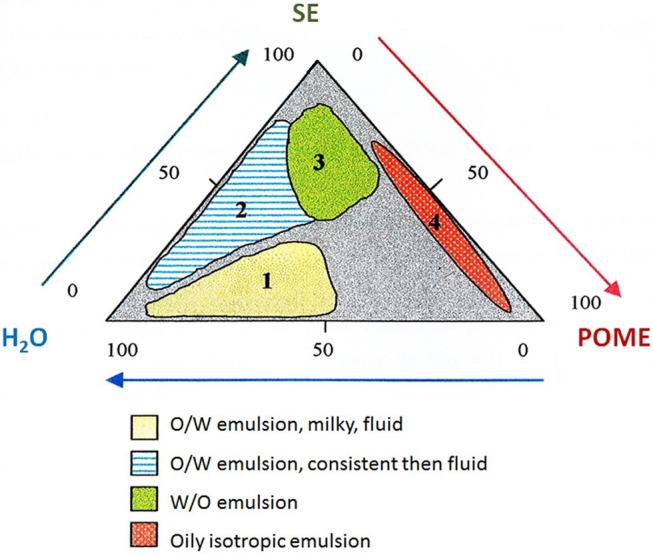
Pseudo-ternary phase diagram for POME/H_2_O/SE mixture. POME: palm oil methyl ester; SE: sucrose ester. Adapted from Claverie (1998).

Claverie et al. described a process for the synthesis of sucrose esters with a low DS using sucrose, palm oil methyl ester (POME), a basic catalyst and another sucrose ester (with a DS 1–3) as a non-ionic surfactant. This synthesis was performed in two main steps. In the first step, the reagents were premixed to prepare the organized medium (catalyst: K_2_CO_3_ (5% by wt, 80–100°C for 15–30 min). The key parameters for this step are the shearing of the medium, the temperature and the composition of the mixture. For this step, a pseudo-ternary-phase diagram of water/POME/sucrose esters was produced, under conditions of mechanical agitation, and the different phases were analyzed under a polarizing microscope. Four zones were identified on the diagram: fluid milky O/W emulsions, consistent and fluid O/W emulsions, W/O emulsions and oily and isotropic emulsions with droplets of more than 100 μm in diameter (Figure 1). The emulsions were stabilized and contact between the reagents was increased by decreasing the particle size through a change in shearing method and by studying the impact of the catalyst. Agitation with a rotor/stator stirrer greatly decreased particle size and, in the presence of micronized K_2_CO_3_, the aggregates were 30 μm in diameter, regardless of the zone (Table 1). This study showed that a stable milky, fluid O/W emulsion promotes the reaction. It also revealed that preferential dispersion of the micronized K_2_CO_3_ catalyst in the fatty phase made a major contribution to reactivity due to the irreversible adsorption of this substrate. Under high-pressure homogenization (400 bars), aggregates of 1 μm in diameter were obtained.

**Table 1:** Size of the droplets of sucrose ester emulsions.

**Zone**	**Mechanical agitation 1,000 t/min**	**Rotor/stator 3,000 t/min**	**K_**2**_CO_**3**_ + Rotor/stator 3,000 t/min**
1	>100 μm	15 μm	30 μm
2	>100 μm	5–10 μm	30 μm
3	>100 μm	30 μm	30 μm
4	>100 μm	30 μm	30 μm

The sucrose esters were then formed. The premix was loaded into a reactor and transesterification was allowed to occur at 120–140°C, 2–8 mbar for 4 h. During this process, the transesterification reaction of acyl transfer was coupled with the continuous elimination of methanol. The proposed mechanism of sucrose esters synthesis is as follows: in the first step, the reagents are adsorbed onto the surface of the catalyst, where the reaction takes place. The carbonyl group is activated by the delocalization of its π electrons (Mouloungui et al., 1989). The reaction is therefore favored at the surface, and then at the interface, where the hydroxyl group of a sucrose molecule can react easily. The presence of sucrose esters during this emulsion/dispersion step helps to stabilize the medium. The next step was the desorption of the reaction products. This study revealed that the reaction involves the generation of an intermediate with a DS of 6, with acyl transfer from this intermediate to sugar. Monoesters (15%), diesters (25%) triesters and higher order esters (60%) with a DS of between 1 and 2 were obtained (Claverie et al., 2004).

Zhao et al. synthesized sucrose esters under anhydrous conditions by basic heterogeneous catalysis in the presence of methyl esters as acyl donors. They studied the solubility of sucrose in methyl esters with and without the addition of fatty acid salts or sucrose esters as surfactants. They found that the use of these compounds increased sucrose solubility and resulted in the generation of emulsion. The mechanism of sucrose esters synthesis consisted of three main steps. First, 0.112 moles of sucrose and 0.335 moles of methyl stearate were mixed in a vacuum system at 40°C, dehydrated and deacidified with calcium oxide. The temperature was increased to 120°C and a mechanical mixer operating at 1,000 rpm was used to mix the reagents. The sugar and ester were then adsorbed onto the surface of the sodium hydroxide used as a catalyst (previously dried), leading to the formation of a sucrose monoester. The sucrose ester formed was desorbed. The sucrose ester can facilitate sugar dissolution and undergo additional adsorption and desorption steps to generate polyester. An analysis of the products by high-performance liquid chromatography revealed the presence of mono-, di- and tri-esters (Zhao et al., 2014).

The performance of reactions in emulsions makes it possible to develop organized molecular systems. The use of emulsions also facilitates the control over the DS. Indeed, the studies described above have shown that the mode of shearing affects the process of emulsion formation and can be modulated to obtain small droplets and increase contact between reagents. Sugar esters produced in emulsions are suitable for use in the pharmaceutical, food, and cosmetic industries.

## Conclusions

The synthesis of sugar fatty acid esters has been the focus of considerable research efforts. Biosourced products are at the heart of major economic and industrial challenges, and, with the development of environmental regulatory constraints, the production and consumption of biosourced products have recently increased in response to strong consumer demand. This trend is opening up new opportunities for the development of plant chemistry. This review focuses on efficient and clean methods for synthesizing sugar fatty acid esters. Sugar fatty acid esters can be synthesized with ionic liquids and deep eutectic solvents as solvents and/or catalysts, or by an emulsification process. ILs and DESs are promising solvents for the synthesis of SFAEs because they can be used to dissolve a wide range of compounds of different polarities. However, the use of ILs as solvents remains controversial, due to the reagents used and their method of preparation, together with uncertainties about their biodegradability. DESs are genuine green alternative solvents to organic solvents and ILs, as they are obtained from natural and renewable raw materials. Their properties could be improved because they are potentially limiting for use in the synthesis of sugar esters. For the emulsification process, control over the emulsification steps (dispersion by appropriate techniques) has proved essential for efficient acyl transfer reactions and withdrawal of the co-product. Sugar esters with a low degree of substitution can be obtained if the reactions conditions are controlled appropriately.

The use of a DES organized medium for the synthesis of SFAEs could effectively comply with the principles of green chemistry. Indeed, the syntheses are based on non-toxic methods with no deleterious consequences to the environment thanks to the use of renewable raw materials, safe solvents, catalytic and non-stoichiometric reagents. The use of DESs constitutes a real improvement in terms not only of energy conservation, but also in terms of atom sparing and the generation of a smaller chemical footprint.

## Author Contributions

All authors listed have made a substantial, direct and intellectual contribution to the work, and approved it for publication.

### Conflict of Interest Statement

The authors declare that the research was conducted in the absence of any commercial or financial relationships that could be construed as a potential conflict of interest.

## Abbreviations

DS: degree of substitution
DES: deep eutectic solvent
ILs: ionic liquids
SEs: sucrose esters
SFAEs: sugar fatty acid esters.

## Glossary

Degree of substitution (DS): the number of esterified hydroxyl groups on a sugar.

Hydrophilic-lipophilic balance (HLB): it is the balance between contributions (size and strength) of the hydrophilic and lipophilic moieties of a surfactant molecule.

Critical micelle concentration (CMC): it is defined as the concentration of surfactants above which additional surfactants added to the system form micelles). O/W emulsion: oil-in-water emulsion (oil droplets are dispersed in a continuous water phase). W/O emulsion: water-in-oil emulsion (water droplets are dispersed in a continuous oil phase).

Emulsifying stability: can be defined as the system's ability to resist changes (coalescence, flocculation) over time.

Foaming ability: it is defined as the capacity of the surfactants to form foam irrespective of the special foam properties.

Foam stability: it is determined with the measure of the variations of foam height or volume with time, immediately after foam generation. Foaming ability and foam stability are interrelated.
