# Supplementary material for: Atypical Reaction Media and Organized Systems for the Synthesis of Low-Substitution Sugar Esters
Source: Front Chem. 2019 Sep 23;7:587. doi: 10.3389/fchem.2019.00587 (PMC6768285; doi:10.3389/fchem.2019.00587)
Supplement: Supplementary file 1 [file Table_1.DOCX]

Supplementary Material


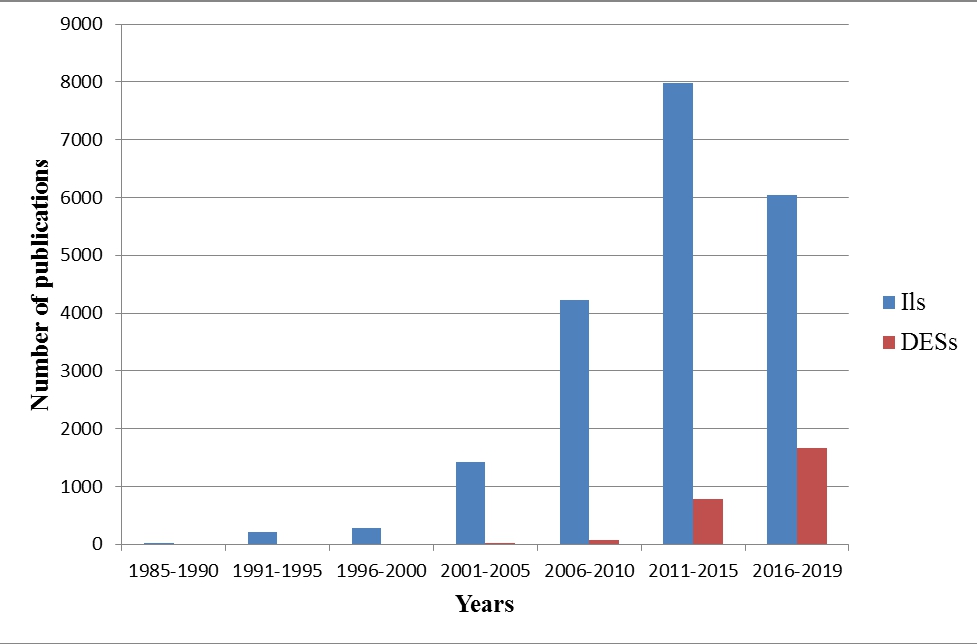


Figure 1: Changes in the number of publications reporting the use of ILs and DESs as solvents

Table 1 : Salts and hydrogen bond donors (HBD) involved in the formation of DES.

| Salts | Hydrogen bond donors (HBD) |
| --- | --- |
|    Choline chloride Betaine    Choline acetate |     Urée Acetamide Glycerol     Ethylene glycol Glucose |

Note : Different Salt : HBD ratios are used (1:1, 1:2, 2:1) - (Pöhnlein et al., 2015; Zhao et al., 2016).

Table 2 : Composition of different ionic liquids for the synthesis of sugar fatty acid esters

| ILs | Cations | Anions | | References |
| --- | --- | --- | --- | --- |
| [Bmim][dca]  [Bmim][BF_4_]  [Bmim][PF_6_]  [Bmim][TFO]  [Bmim][TF_2_N] |   1-Butyl-3-methylimidazolium | Dicyanamide    Tetrafluoroborate    Hexafluorophosphate  Trifluoromethanesulfonate  Bis(trifluoromethylsulfonyl)imide |          | (Forsyth and R. MacFarlane, 2003; Liu et al., 2005; MacFarlane et al., 2001)  (Ganske and Bornscheuer, 2005)  (Findrik et al., 2016; Ganske and Bornscheuer, 2005)  (Liang et al., 2012; Mai et al., 2014)  (Mai et al., 2014) |
| [Hmim][TFO] |   1-Hexyl-3-methylimidazolium | Trifluoromethanesu lfonate |  | (Lin et al., 2015) |
| [Bu_4_N][Ac]  [Et_4_N][Ac]  [Me_4_N][Ac] |   Tetraalkylammonium  (R=butyl, ethyl, methyl) | Acetate |  | (Lin et al., 2016) |

Table 3 : Population and size of globules of a POME/H2O/SE/K2CO3 mixture in a high-pressure homogenizer (Claverie et al., 2004)

| Pressure | Droplet size (µm) | Apparatus |
| --- | --- | --- |
| Atmospheric | 15 µm | Polarizing microscope |
| 200 bar | 3 µm | Polarizing microscope |
| 400 bar | 1 µm | Polarizing microscope |
| 900 bar | 2 populations:  (60 % at 0,3 µm and 32 % at 0,5 µm) | Light scattering (Nanosizer) |

**
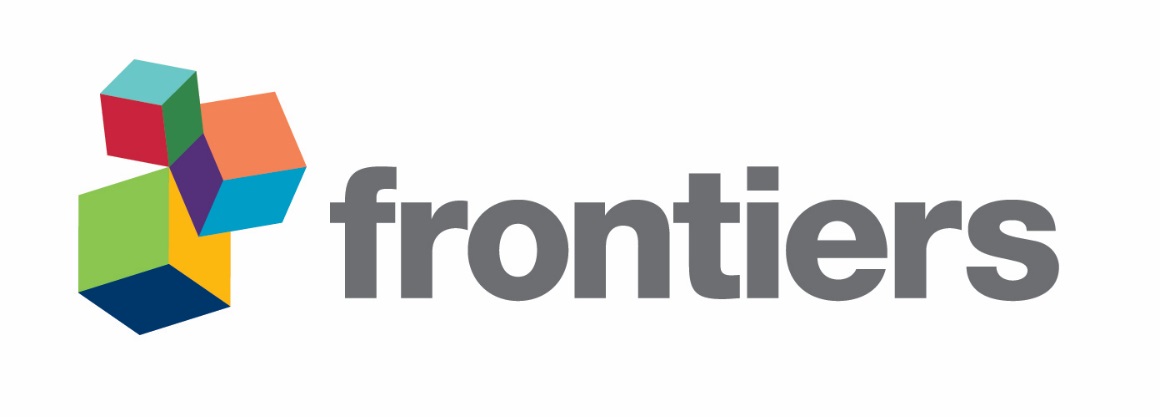
**
